# Supplementary material for: The application of resilience assessment grid in healthcare: A scoping review
Source: PLoS One. 2022 Nov 4;17(11):e0277289. doi: 10.1371/journal.pone.0277289 (PMC9635744; doi:10.1371/journal.pone.0277289)
Supplement: S1 Appendix — (DOCX) [file pone.0277289.s001.docx]

**Preferred Reporting Items for Systematic reviews and Meta-Analyses extension for Scoping Reviews (PRISMA-ScR) Checklist**

A protocol for the scoping review was published in the Open Science Framework (OSF)( Registration DOI. [10.17605/OSF.IO/GTCZ3](https://doi.org/10.17605/OSF.IO/GTCZ3)). The protocol and this checklist are the same.

| **SECTION** | **ITEM** | **PRISMA-ScR CHECKLIST ITEM** | **REPORTED ON PAGE #** |
| --- | --- | --- | --- |
| **TITLE** | | | |
| Title | 1 | The application of resilience assessment grid in healthcare: A scoping review | 1 |
| **ABSTRACT** | | | |
| Structured summary | 2 | Abstract provided | 2-3 |
| **INTRODUCTION** | | | |
| Rationale | 3 | The review will provide a comprehensive description of the state-of-the-art application of the RAG in healthcare to practitioners and academics. | 3-4 |
| Objectives | 4 | The aim of this scoping review is to understand how the RAG method has been applied into practice in healthcare and what outcomes have been attributed to its application. With a view to assisting practitioners and decision-makers as well as researchers, we set out to clarify:   - What methods are used to adapt the Resilience Assessment Grid? - How has the Resilience Assessment Grid been put into practice and in which settings? - What outcomes have been attributed to Resilience Assessment Grid application in healthcare? | 3-4 |
| **METHODS** | | | |
| Protocol and registration | 5 | A protocol for the scoping review was published in the Open Science Framework (OSF)( Registration DOI. [10.17605/OSF.IO/GTCZ3](https://doi.org/10.17605/OSF.IO/GTCZ3)). | 5 |
| Eligibility criteria | 6 | We intend to capture all empirical research on RAG in heathcare: The inclusion criteria are studies from the healthcare setting, reporting on empirical research on RAG, published in peer-reviewed journals or books, as well as unpublished reports. These will be restricted to English language. No limitations are set regarding publication year. The search was conducted in [April 2021]. | 5 |
| Information sources* | 7 | A specialist librarian assisted with search strategy. The databases systematically searched were; Medline, Embase and Web of Science.  Supplementary search were:   - Cited paper – Erik Hollnagel’s paper on RAG - Google scholar - Book chapters: resilience engineering books - Resilient Health Care Net (RHCN) website: https://resilienthealthcare.net/about/ | 6 |
| Search | 8 | Medline and Embase:  (resili* adj3 (grid or engineering or assessment or analysis)) | 6 |
| Selection of sources of evidence† | 9 | All the selection steps will be performed by 3 reviewers independently of each other. One of the reviewer will review all the titles and abstracts. The other 2 reviewer will divide retrived studies between them. - Discrepancies between the reviewer’s selection results, would be discussed with a third researcher until consensus on inclusion or exclusion was reached. The Covidence software program would be used in the study selection process. | 7 |
| Data charting process‡ | 10 | An excel spreadsheet will be specifically designed for this review and will be piloted by one reviewer.  3 reviewers will independently of each other extract data. One of the reviewer will extract the data from all the studies. The other 2 reviewer will divide randomly the retrived studies between them. Lastly, the extracted data will be reviewed by the three reviewers, and disagreements will be discussed in the group. | 7 |
| Data items | 11 | Data to be extracted will include, but may not be limited to: First Author, Title, Year of publication, Country, Setting, objective, target group, publication type, study design, methods used to adapt RAG, how was RAG applied measure resilience performance, outcome of RAG | 7-8 |
| Critical appraisal of individual sources of evidence§ | 12 | N/A | Click here to enter text. |
| Synthesis of results | 13 | A narrative synthesis will be performed. It will describe the scope of existing research and summarized data using structured narrative and summary tables. Data will be presented in tabular and graphical presentation of the included studies. | 7-8 |
| **RESULTS** | | | |
| Selection of sources of evidence | 14 | See the provided Prisma flow-diagram | Click here to enter text. |
| Characteristics of sources of evidence | 15 | See table 2. | Click here to enter text. |
| Critical appraisal within sources of evidence | 16 | N/A | Click here to enter text. |
| Results of individual sources of evidence | 17 | See the result section | 8-14 |
| Synthesis of results | 18 | See the result section | Click here to enter text. |
| **DISCUSSION** | | | |
| Summary of evidence | 19 | See discussion section | 14-17 |
| Limitations | 20 | Although only English language articles were included in the review, some of these reported on studies where the RAG was applied in languages other than English. | 17 |
| Conclusions | 21 | The RAG has been applied in a variety of healthcare settings and is a promising tool to manage some of the current and future challenges of the healthcare system. To better realise the potential benefits of the RAG, it is important that we move beyond the development phase of the RAG tool to implementing quality initiatives and managing them. | 17-18 |
| **FUNDING** | | | |
| Funding | 22 | This research is part of a ph.d. project and funded by University Hospital of Southern Denmark and University of Southern Denmark. | 18 |

JBI = Joanna Briggs Institute; PRISMA-ScR = Preferred Reporting Items for Systematic reviews and Meta-Analyses extension for Scoping Reviews.

* Where *sources of evidence* (see second footnote) are compiled from, such as bibliographic databases, social media platforms, and Web sites.

† A more inclusive/heterogeneous term used to account for the different types of evidence or data sources (e.g., quantitative and/or qualitative research, expert opinion, and policy documents) that may be eligible in a scoping review as opposed to only studies. This is not to be confused with *information sources* (see first footnote).

‡ The frameworks by Arksey and O’Malley (6) and Levac and colleagues (7) and the JBI guidance (4, 5) refer to the process of data extraction in a scoping review as data charting*.*

§ The process of systematically examining research evidence to assess its validity, results, and relevance before using it to inform a decision. This term is used for items 12 and 19 instead of "risk of bias" (which is more applicable to systematic reviews of interventions) to include and acknowledge the various sources of evidence that may be used in a scoping review (e.g., quantitative and/or qualitative research, expert opinion, and policy document).

*From:* Tricco AC, Lillie E, Zarin W, O'Brien KK, Colquhoun H, Levac D, et al. PRISMA Extension for Scoping Reviews (PRISMAScR): Checklist and Explanation. Ann Intern Med. 2018;169:467–473. [doi: 10.7326/M18-0850](http://annals.org/aim/fullarticle/2700389/prisma-extension-scoping-reviews-prisma-scr-checklist-explanation).
