# Supplementary material for: The application of resilience assessment grid in healthcare: A scoping review
Source: PLoS One. 2022 Nov 4;17(11):e0277289. doi: 10.1371/journal.pone.0277289 (PMC9635744; doi:10.1371/journal.pone.0277289)
Supplement: Attachment — Submitted filename: Response to Reviewers_ RAG ScR PLOS ONE.docx [file pone.0277289.s002.docx]

3^rd^ June 2022

Dear Editor and reviewers,

On behalf of my co-authors, many thanks for taking the time to provide constructive comments and enabling us to submit a revised manuscript.

We believe these revisions have resulted in a significantly improved manuscript. We outline in detail how we have handled each of the reviewers’ comments and the additional journal requirements below.

We look forward to hearing back from you.

Best regards,

Mariam Safi on behalf of all the co-authors

**Response to Journal requirements:**

1. Formatted the manuscript to meet the PLOS ONE’s style requirements (figures, tables, headings etc.). Used PACE to format the figures.
2. Tables and figures were added to the main manuscript.
3. There is no grant number associated with the funding support.
4. Funding statement is included in the cover letter above.
5. Role of Funder statement is included in the cover letter above.
6. Competing interest. Adherence to all PLOS ONE policies on sharing data is confirmed in the aforementioned cover letter.
7. Bewick et al. study. This study was not referenced in our paper.
8. Table 3 was labelled as Table 4 in error. This has been corrected – line 305.
9. Supporting information files updated. Please see lines 95 and 429-430.
10. Reviewed the reference list. We believe no changes are needed.

**Response to reviewers’ comments:**

| **Reviewer 1 comments** | **Response** |
| --- | --- |
| Over all comment- spacing issues with respect to putting references in the text(numbers and et.al) | Thank you for the comment. We have looked through the manuscript and eliminated unnecessary spacing. |
| line 33- start the sentence with 12 in words not number. | Thank you for pointing this out. We have made the change, please see line 33. |
| line 68-reference missing | We have added the missing references – Line 67. |
| line 102-May 21 needs to be in sentence, to make more sense. | Thank you for the comment. Please see line 100 for the revision. |
| line 242-it should be- actors | Thank you. Please see line 281 for the amendment. |
| line 247- it should be- conducted in the anesthesia area. | Thank you. The extra “in” is deleted now. Please see line 286. |
| line 282- omit- otherwise | Thank you for noticing it. We have deleted “otherwise” – line 321. |
| line 343- replace is with are | Thank you. Please see line 381 for the change. |
| **Reviewer 2 comments** | **Response** |
| A methodologically sound review paper. The authors have addressed an important issue in appraising resilience in health care settings. Like most issues which are important, resilience is difficulty to quantify. It will also be influenced by different settings and cultures. The present study can be considered very preliminary and the concepts need to be validated in different settings. | Thank you for your support of our paper. We agree that assessing an organization’s potential to perform resiliently can be very difficult to quantify. As you mentioned, resilient performance is context dependent and we have suggested in the discussion and conclusion section that RAG needs to be validated in different settings. |
