# Supplementary material for: The application of resilience assessment grid in healthcare: A scoping review
Source: PLoS One. 2022 Nov 4;17(11):e0277289. doi: 10.1371/journal.pone.0277289 (PMC9635744; doi:10.1371/journal.pone.0277289)
Supplement: Attachment — Submitted filename: Response to Reviewers.docx [file pone.0277289.s003.docx]

15 July 2022

Dear Editor and Reviewers,

Thank you again for carefully reading our manuscript. We also appreciate your suggestions, which have been very helpful in improving the manuscript.

All your comments have been taken into account. In the following table, we present in detail how we have handled each comment. We have used tracked changes to indicate the amendments in the manuscript.

We hope that these changes to the manuscript will facilitate the decision to publish this study in PLOS ONE.

We look forward to hearing back from you.

Best regards,

The authors

| **Reviewer 1 comments** | **Response to reviewer** |
| --- | --- |
| Line 24:Following sentence can be reframed as: This scoping review aims to understand the practical application of RAG method and its outcomes in healthcare. | Thank you. The sentence has been reframed. Please see line 24-25. |
| Line 33: add "the": met the inclusion criteria. reframe the next sentence, in 2 sentences: Diversities were found across study designs (instead of design) and methodologies(instead of methods). Qualitative designs(instead of methods)and literature reviews...practice. | Thank you, please see line 33- 34. |
| Line 35:replace "apply" with applied it in practice. | Thank you, please see line 35. |
| Line 42- omit "better" | Thank you, please see line 42. |
| Line 43: replace " to using it to" with "and use it to"....initiatives. | Thank you, please see line 43. |
| Line 56: replace "sets" with "set" | Thank you, please see line 56. |
| Line 82, 83,84: use abbreviation- RAG | Thank you, please see line 82 – 84. |
| Line 97:reframe this sentence-"The inclusion criteria was to select studies from.....reports". | Thank you, please see line 97. |
| Line 120 It should be- "as per advice of the...librarian". | Thank you, please see line 120. |
| Line 127: it should be-"after removal of the duplicates in Endnote,...." | Thank you, please see line 128. |
| Line 155: reframe this sentence as -"after removal of duplicates,1365 citations were retained". | Thank you, please see line 156-157. |
| Line 156: reframe this as- "After the full text assessment, 12 articles were included and 31 articles were excluded. comment-we should always mention inclusions first . | Thank you, the sentence has been reframed. Please see line 158. |
| Line185: add "onwards"-from 2018 onwards. | Thank you, please see line 187. |
| Line 188- it should be- "in" intensive care unit instead of an. | Thank you, please see line 190. |
| Line 189: it should be-"Eight studies employed.....".instead of eight of the studies.... | Thank you, please see line 191. |
| Line 191: reframe sentence as-"All studies employed a case study design except one study(14) which had cross sectional design". | Thank you, please see line 193 -194. |
| Line194: it should be-"Eleven studies were prospective". | Thank you, please see line 196. |
| Line 265: pls add" items" in the end of sentence after 3. | Thank you, please see line 267. |
| Line 280- it should be- one of the study instead of studies. | Thank you, please see line 282. |
| Line 336: reframe it as-"A solution to this can be.....again". | Thank you, please see line 338. |
| Line 338: it should be: "were" instead of "are" heterogenous | Thank you, please see line 340. |
| Line 355-357: Its a very long sentence. It can be reframed as- "While all organizations (plural)..... many organizations(plural).....................to the activities(plural)". "However not all........resilient". | Thank you, the sentence has been reframed. Please see line 356-360. |
| Line 379:replace" is" with "are". | Thank you, please see line 381. |
| Line 380: unclear sentence to me after "and". Please reframe it for better understanding. | Thank you for pointing this out. The sentence has be reframed. Please see line 382-383. |
| Line 412-"better " can be omitted | Thank you, please see line 414. |
