# Supplementary material for: The application of resilience assessment grid in healthcare: A scoping review
Source: PLoS One. 2022 Nov 4;17(11):e0277289. doi: 10.1371/journal.pone.0277289 (PMC9635744; doi:10.1371/journal.pone.0277289)
Supplement: Attachment — Submitted filename: Response to reviewers.docx [file pone.0277289.s004.docx]

27^th^ September 2022

Dear Editor and Reviewers,

Thank you again for carefully reading our manuscript. We also appreciate your suggestions, which have been very helpful in improving the manuscript.

All your comments have been taken into account. In the following table, we present in detail how we have handled each comment. We have used tracked changes to indicate the amendments in the manuscript.

We hope that these changes to the manuscript will facilitate the decision to publish this study in PLOS ONE.

We look forward to hearing back from you.

Best regards,

The authors

**Response to reviewer’s comments:**

| **Reviewer 1 comments** | **Response** |
| --- | --- |
| General comment-please leave a space after putting reference number brackets, follow the same throughout the article for uniformity. | Thank you for the comment. We amended the reference layout throughout the article. |
| Line59-it should be- add are-"original questions that are based on....." | Thank you. Please see line 59. |
| Line 65- replace "that" with "which" to make sentence sound better. "which recognises that......" | Thank you for your advice. We have run your suggestion past one of our coauthors who is a native English speaker and believe that the original is correct.(line 64-65). |
| Line 75- replace academics with academicians | Thank you for your advice. We have run your suggestion past one of our coauthors who is a native English speaker and believe that the original is correct. |
| Line 100- sentence should be better framed-"The articles were excluded...." as- "The articles that described resilience assessment grids in non-healthcare settings, resilience papers not using RAG, individual or community resilience, conference abstracts with no full texts, or were theoretical/review papers were excluded. | Thank you the sentence has been reframed. Please see lines 100-102. |
| Line 101-what is meant by individual or community resilience, it looks incomplete to me. Please reframe it. | Thank you for the comment. We have explained it further in lines 103-104. |
| Line 136- it should be the excel instead of Excel | Thank you. Excel is the name of the program, so the capitalisation is correct in this instance. We have corrected the name to Microsoft Excel to clarify. |
| Line 143-"The second and the third reviewer..." it should be. | Thank you for your advice. We have run your suggestion past one of our coauthors who is a native English speaker and believe that the original is how it is normally written. |
| Line 235- It should be allocation instead of allocations | Thank you – we have made the correction. Please see line 252. |
| Line 253-it should be -"psychological safety and problem solving". | Thank you– we have made the correction. Please see line 271. |
| Line 286- it should be anesthesia domain or department or unit. Please check original article, and replace with suitable option. | Thank you, please see line 288. |
| Line 301- replace "overview over.....".with "overview about...." | Thank you – we have made a number of corrections to improve the flow of this paragraph. Please see lines 319-324. |
| Line 355- replace "many organization..." with "many organizations..." | Thank you. Please see line 378-380. |
| Line 389- it can be better framed as-"healthcare still evolving, evidenced by........2018". | Thank you. Please see line 415. |
| Line 390- omit and after however | Thank you. Please see line 416. |
| Line 413-omit RAG with "it"-as its repeating | Thank you. Please see line 440. |
