# Supplementary material for: The application of resilience assessment grid in healthcare: A scoping review
Source: PLoS One. 2022 Nov 4;17(11):e0277289. doi: 10.1371/journal.pone.0277289 (PMC9635744; doi:10.1371/journal.pone.0277289)
Supplement: Attachment — Submitted filename: Response to reviewers_v4.docx [file pone.0277289.s005.docx]

24^th^ October 2022

Dear Editor and Reviewers,

Thank you again for carefully reading our manuscript. We also appreciate your suggestions, which have been very helpful in improving the manuscript.

All your comments have been taken into account. In the following table, we present in detail how we have handled each comment. We have used tracked changes to indicate the amendments in the manuscript.

We hope that these changes to the manuscript will facilitate the decision to publish this study in PLOS ONE.

We look forward to hearing back from you.

Best regards,

The authors

**Response to reviewer’s comments:**

| **Reviewer 1 comments** | **Response** |
| --- | --- |
| Line 255-256: we can omit Chuang et.al from the sentence "In the first study.....an expert panel", as the sentence prior to it mentions that Chuang et .al did two related studies. | Thank you for the comment. We have made the correction. Please see line 256. |
| Line 260: pls put space after the bracket closes "(SNA) to" | Thank you. Please see line 260 for the change. |
